# In vivo magnetic resonance spectroscopy by transverse relaxation encoding with narrowband decoupling

**DOI:** 10.1038/s41598-023-39375-0

**Published:** 2023-07-27

**Authors:** Li An, Jun Shen

**Affiliations:** 1 Molecular Imaging Branch, National Institute of Mental Health, National Institutes of Health, Bethesda, MD USA; 2 Molecular Imaging Branch, National Institute of Mental Health, National Institutes of Health, Building 10, Room 3D46, 10 Center Drive, MSC 1216, Bethesda, MD 20892-1216 USA

**Keywords:** Imaging, Molecular imaging

## Abstract

Cell pathology in neuropsychiatric disorders has mainly been accessible by analyzing postmortem tissue samples. Although molecular transverse relaxation informs local cellular microenvironment via molecule-environment interactions, precise determination of the transverse relaxation times of molecules with scalar couplings (J), such as glutamate and glutamine, has been difficult using in vivo magnetic resonance spectroscopy (MRS) technologies, whose approach to measuring transverse relaxation has not changed for decades. We introduce an in vivo MRS technique that utilizes frequency-selective editing pulses to achieve homonuclear decoupled chemical shift encoding in each column of the acquired two-dimensional dataset, freeing up the entire row dimension for transverse relaxation encoding with J-refocusing. This results in increased spectral resolution, minimized background signals, and markedly broadened dynamic range for transverse relaxation encoding. The in vivo within-subject coefficients of variation for the transverse relaxation times of glutamate and glutamine, measured using the proposed method in the human brain at 7 T, were found to be approximately 4%. Since glutamate predominantly resides in glutamatergic neurons and glutamine in glia in the brain, this noninvasive technique provides a way to probe cellular pathophysiology in neuropsychiatric disorders for characterizing disease progression and monitoring treatment response in a cell type-specific manner in vivo.

## Introduction

Magnetic resonance spectroscopy (MRS) enables noninvasive detection of molecules in vivo, including those found in the highly inaccessible human brain. Neuropsychiatric disorders and brain tumors are among the most active areas of in vivo MRS research. Accurate in vivo detection of key neurochemicals such as glutamate (Glu), glutamine (Gln), and glutathione (GSH) is highly important in these fields. Glu is the primary excitatory neurotransmitter in the central nervous system (CNS). The metabolic coupling between Glu and Gln forms the Glu-Gln neurotransmitter cycle between glutamatergic neurons and glia^1,2^. Glu and Gln are also major metabolites in the CNS, playing crucial roles in normal brain function and cancer cell growth^3,4^. GSH is an antioxidant, whose level is a marker of redox state^5^. Glu, Gln, and GSH have been implicated in many illnesses such as epilepsy, schizophrenia, bipolar disorders, Alzheimer’s disease, major depressive disorder, anxiety disorders, and brain tumors^6–11^. Many brain disorders are also associated with alterations in cell type-specific microenvironments^12^, which are difficult to measure noninvasively. For instance, postmortem studies have found ample evidence of glial pathology in major depressive disorder^13,14^ and one of the defining characteristics of the pathophysiological state of Alzheimer’s disease is the atrophy of glutamatergic neurons^15^.

For the majority of in vivo single-voxel MRS experiments, localized one-dimensional free induction decay (FID) signals are acquired with a large number of transients to achieve sufficient signal-to-noise ratio (SNR). Chemical shift information is frequency-encoded in the FIDs, along with signal modulation due to J-coupling. The frequency domain MRS spectrum is obtained by Fourier transforming the sum of all transients. Signal modulation due to J-coupling causes peak splitting in an MRS spectrum, which can impair quantification by reducing peak amplitudes and increasing spectral overlap among different molecules and with the background signals (macromolecule signals and the background spectral baseline). Recent MRS studies of the human brain have revealed errors and uncertainties in modeling background signals in the widely used short echo time (TE) spectra^16–19^.

In addition to measuring concentrations, in vivo MRS can also determine transverse relaxation times (T_2_)^20–24^. Unlike the T_2_ of tissue water measured by magnetic resonance imaging (MRI), the molecular T_2_s measured by MRS are often cell type-specific and can provide critical information on the cellular microenvironment where the molecules reside through molecule-local environment interactions^25–29^. For instance, in the brain, Glu and Gln are predominantly localized in glutamatergic neurons and glia, respectively. Reliable detection of Glu and Gln T_2_s can therefore provide unique information on the microenvironments of these different cell types in vivo.

There are several inherent difficulties in reliably measuring T_2_ of J-coupled molecules: (i) the signal intensity of many J-coupled molecules is low, partly due to peak splitting caused by J-couplings; (ii) due to J-evolution and spectral overlap, the optimal TE for reliable detection of a J-coupled molecule is fixed^23,30,31^, while T_2_ measurements require a series of very different time points for transverse relaxation encoding; (iii) the background signals also change with TE^32^, and the uncertainty and errors in modeling the background signals lead to significant errors in T_2_ measurements.

Compared to one-dimensional (1D) MRS, two-dimensional (2D) MRS techniques^33–37^ offer many unique capabilities. For example, effective homonuclear decoupling can be achieved in the context of 2D MRS to significantly improve spectral resolution in vivo^34,38–42^. Among them, the 2D ProFit technique has been used to estimate neurochemical T_2_s^43–45^.

In this work, we introduce a novel 2D MRS technique, Transverse Relaxation Encoding with Narrowband Decoupling (TREND), for in vivo measurement of neurochemical concentrations and T_2_ relaxation times. By using frequency-selective editing pulses, this technique achieves chemical shift encoding with selective homonuclear decoupling in the column (t_1_) dimension and T_2_ encoding with single or double J-refocusing in the row (t_2_) dimension. This technique addresses the difficulties in measuring the concentrations and T_2_s of J-coupled molecules by: (i) selectively decoupling the targeted spins in the column dimension; (ii) allowing T_2_ encoding at many time points with markedly broadened dynamic range; (iii) utilizing relatively long TEs and, to a lesser extent, the TE averaging effect^46,47^ to minimize the background signals. As an application of this technique, the concentrations and T_2_ relaxation times of Glu, Gln, and GSH–three important biomarkers for neuropsychiatric and brain cancer research–are measured in the human brain in vivo.

## Results

**Figure 1:**
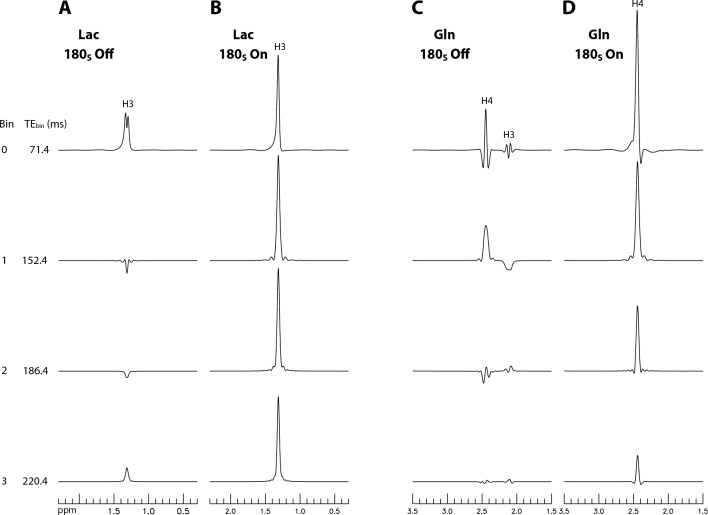
Numerically calculated bin spectra of Lac (**A**,**B**) and Gln (**C**,**D**). The pulse sequence used is shown in Fig. 8 in the “Methods” section. Each stack plot displays a subset of the 11 bin spectra obtained. The dual-band editing pulses (denoted as 180_S_) had two 180° bands, one at 2.12 ppm and the other at 4.10 ppm. The editing pulses were turned off in (**A**) and (**C**) and on in (**B**) and (**D**). The [Lac]:[Gln] ratio was 1:7. TE_bin_ represents the average TE of each bin. *Lac* lactate, *Gln* glutamine.

Density matrix simulated^48,49^ bin spectra of lactate (Lac) and Gln are shown in Fig. 1. For each molecule, two sets of bin spectra were calculated with the frequency-selective editing pulses switched off and on, respectively. Since there is no reported T_2_ value for Lac in the healthy human brain, the T_2_ of Lac was set to 180 ms, similar to that of Glu in the grey matter^23^. T_2_ of Gln was set to 80 ms^23^. All bin spectra were line broadened to a typical in vivo linewidth of 9 Hz. Without the editing pulses (Fig. 1A), the Lac peak is a doublet with low amplitude for bin 0 and becomes even lower for bins 1 – 3 due to its H2-H3 J-evolution. In contrast, when the editing pulses are turned on (Fig. 1B), the frequency band at 4.10 ppm locks the H2-H3 J-evolution in the column (t_1_) dimension. As a result, the Lac H3 peak becomes a sharp singlet in the column dimension with much higher amplitude in all four bin spectra. Gln has a more complex spin system with a strong internal coupling between its two H4 protons but weak external couplings between its H3 and H4 protons^50^. Without the editing pulses, the Gln H4 signal is greatly diminished due to J-evolution and signal self-cancelation (Fig. 1C). By applying the editing pulses to the H3 resonances of Gln at 2.12 ppm, the H3-H4 J-evolutions are locked in the column dimension, resulting in a sharp pseudo singlet for the H4 signal with significantly higher peak intensity (Fig. 1D). Figure 1 shows that the application of the editing pulses resulted in a significant improvement in both spectral resolution and peak amplitude for the targeted spins.

**Figure 2:**
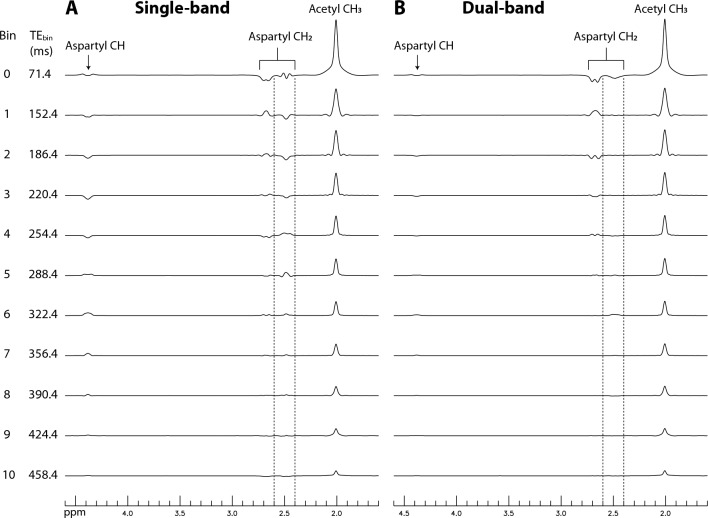
Numerically calculated bin spectra of NAA obtained using single-band (**A**) and dual-band (**B**) editing pulses. The single-band 180° editing pulses were applied at 2.12 ppm. The dual-band editing pulses had a 180° band at 2.12 ppm and a 90° band at 4.38 ppm targeting the NAA aspartyl CH proton. The remaining parameters are provided in Fig. 8 in the “Methods” section. The pair of dashed lines in each stack plot indicate the 2.4–2.6 ppm range, where Gln and GSH glutamyl H4 protons resonate. *NAA* N-acetylaspartate.

Density matrix simulated bin spectra of N-acetylaspartate (NAA) using single-band (A) and dual-band (B) editing pulses are shown in Fig. 2. T_2_ was set to 200 ms for acetyl CH_3_ and 140 ms for aspartyl CH_2_^23^. All bin spectra were line broadened to a typical in vivo linewidth of 9 Hz. In Fig. 2A, the NAA aspartyl CH_2_ resonances at 2.4–2.6 ppm are relatively large and interfere with the detection of Gln and GSH. In contrast, in Fig. 2B, these resonances were effectively suppressed in bins 0–3 due to the second band targeting 4.38 ppm.

**Figure 3:**
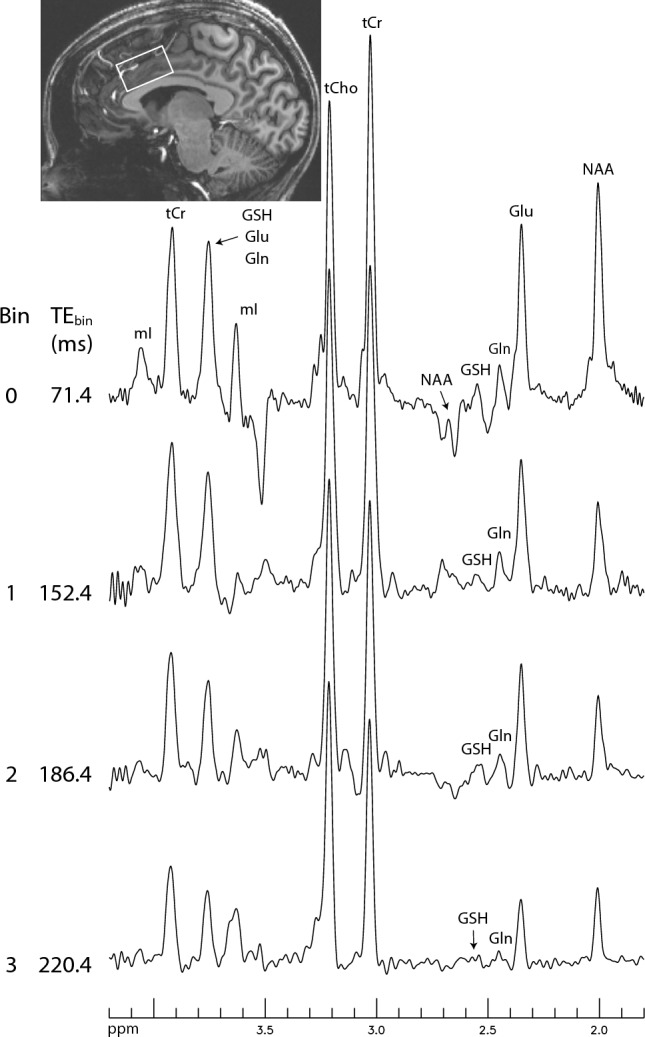
In vivo bin spectra (0–3) acquired from a 2 × 3.5 × 2 cm^3^ voxel in the cingulate cortex of a healthy participant. The pulse sequence used to acquire the in vivo data was the same as that used for Fig. 2B, employing dual-band editing pulses to simultaneously lock the H3-H4 couplings and suppress the NAA aspartyl resonances between 2.4 and 2.6 ppm. No line broadening was applied to the spectra. TE_bin_ represents the average TE of each bin. *mI* myo-Inositol, *tCho* total choline, *tCr* total creatine. The full dataset is shown in Fig. 4.

**Figure 4:**
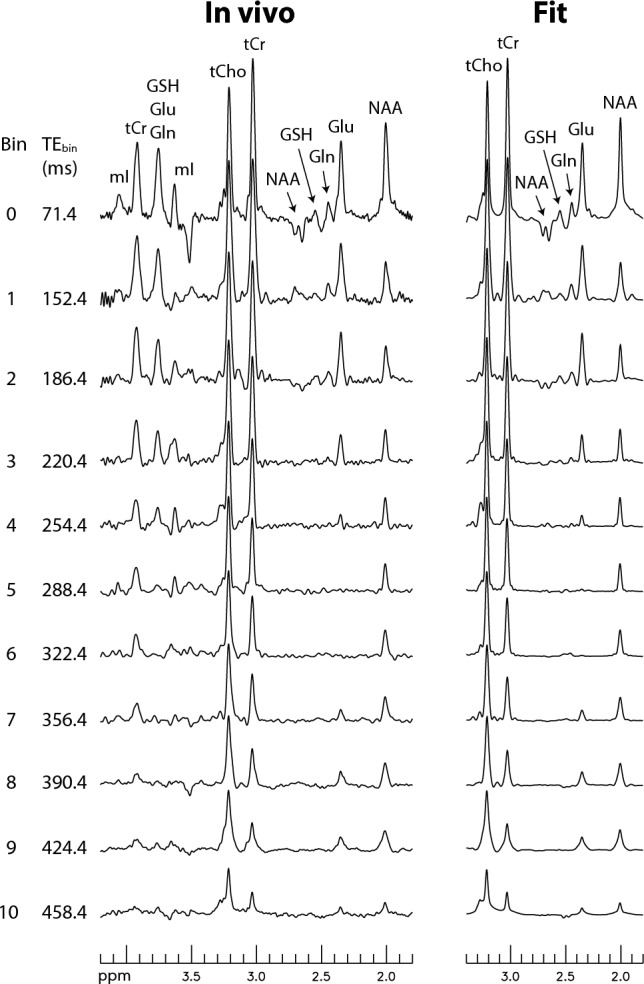
In vivo bin spectra (0–10) and their fits. The entire scan time was 10 min and 43 s using a 2.5 s repetition time (TR).

**Figure 5:**
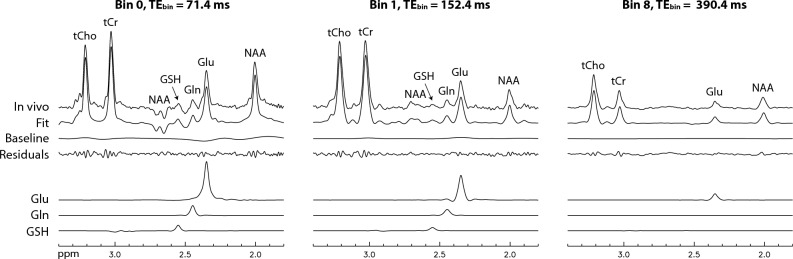
Examples of fitting details for the bin spectra displayed in Fig. 4.

The proposed technique was used to measure the concentrations and T_2_s of Glu, Gln, GSH, total creatine at 3.03 ppm (tCr), and total choline at 3.21 ppm (tCho) in the human brain. Figure 3 shows the first four bin spectra acquired from a healthy participant and the re-test results are provided in the Supplementary Information. The Gln H4 signal appears as a sharp pseudo singlet in the bin spectra, which is consistent with the numerical simulation shown in Fig. 1D. The Gln peak remains detectable in bin 3, which has an average TE (TE_bin_) of 220.4 ms. Figure 4 displays the spectra for all 11 bins and their corresponding fitted spectra. The fitted spectra in the fitting range (1.8–3.4 ppm) closely resemble the in vivo spectra for all 11 bins. The Glu peak appears prominent in bins 0 – 3, which span 71.4–220.4 ms, and is still detectable in bin 10, which has a very long TE_bin_ of 458.4 ms. Figure 5 shows the fitting details for bins 0, 1, and 8. The model spectra fit the in vivo spectra well, as evidenced by the small residuals.

**Figure 6:**
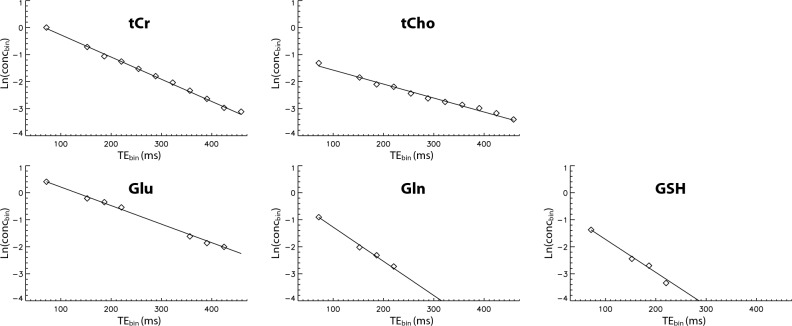
Linear regression of ln(conc_bin_) on TE_bin_. The T_2_-weighted concentrations (conc_bin_) were obtained by fitting the bin spectra shown in Fig. 4.

Linear regressions of ln(conc_bin_) on TE_bin_ for tCr, tCho, Glu, Gln, and GSH are shown in Fig. 6. NAA was not analyzed as it was partially dephased by the 2.12 ppm band of the editing pulses and the crusher gradients. The T_2_-weighted concentrations (conc_bin_) were obtained by fitting the bin spectra shown in Fig. 4. The single exponential model was found to fit the in vivo T_2_ decay data well. The coefficients of determination (R^2^) were greater than 0.95 for tCr, tCho, Glu, and Gln (see Table 1). Table 1 lists the neurochemical concentrations, as ratios to [tCr], and T_2_ relaxation times obtained from the linear regressions.

**Table 1:** Quantification of neurochemical concentrations (1/[tCr]) and T_2_ relaxation times in the cingulate cortex of healthy participants (n = 6; mean ± SD).

	Neurochemical ratio (/[tCr])	CRLB (%)	CV (%)	T_2_ (ms)	CRLB (%)	CV (%)	R^2^
tCr [N(CH_3_)]	1	1.1 ± 0.3	0	116 ± 6	0.6 ± 0.1	2.3	0.99 ± 0.00
tCho [N(CH_3_)_3_]	0.21 ± 0.01	1.2 ± 0.3	2.4	182 ± 12	0.8 ± 0.2	2.7	0.99 ± 0.01
Glu	1.38 ± 0.12	2.1 ± 0.6	5.6	142 ± 11	1.8 ± 0.5	3.9	0.97 ± 0.03
Gln	0.52 ± 0.09	9.3 ± 2.4	7.1	77 ± 8	6.1 ± 1.9	4.0	0.95 ± 0.06
GSH	0.24 ± 0.07	20.8 ± 16.1	16.2	98 ± 17	15.1 ± 11.1	9.6	0.82 ± 0.19

The voxel had 54.5% ± 5.1% grey matter, 37.9% ± 5.0% white matter, and 7.6% ± 4.4% cerebrospinal fluid. The within-subject coefficients of variation (CVs) were calculated from test and re-test measurements of the same voxel.

## Discussion

Cellular pathology in brain disorders has mainly been characterized by postmortem methods^12–15^, which are difficult to standardize due to uncontrollable physicochemical and morphological changes in cells after death, complicating the understanding of disease etiology and development of effective treatment strategies. For example, although numerous postmortem studies have implicated glutamatergic dysregulation in central pathologies, progress in developing therapeutics targeting the glutamatergic system has been relatively slow. This is due, in part, to the lack of tools that can probe and monitor the pathophysiological processes in glutamatergic neurons and glia accompanying disease progression and treatment response.

MRS has high potential for generating cell type-specific contrast in vivo via molecule-environment interactions. However, the precision of measuring T_2_ of J-coupled molecules using previously published MRS technologies has been inadequate. Furthermore, recent methodological re**-**examination of short-TE MRS for neurochemical profiling has found that spectral overlap between neurochemicals and the background signals, as well as between neurochemicals, can result in significant errors in both quantifying neurochemical concentrations and determining correlations between neurochemical concentrations and clinical metrics^16,17,51,52^. These errors propagate into T_2_ measurements that include short-TE spectra. Although neurochemical concentrations measured by spectral fitting of short-TE MRS spectra have been treated implicitly or explicitly as spectrally uncorrelated variables in clinical MRS studies, severe spectral overlap can confound determination of correlations of biological origin^52^.

Alternatively, some 1D in vivo MRS experiments use relatively long TE to minimize the problematic background signals. However, this approach suffers from unknown T_2_ weighting, as changes in T_2_ are associated with many diseases^26,27,29^. Different T_2_ weightings are routinely encountered in clinical MRS literature, leading to many controversies in clinical MRS findings^27^. When the previously published MRS techniques are used to measure molecular T_2_s, the dynamic range for encoding the T_2_s of J-coupled molecules can be very limited. This is because it is necessary to detect J-coupled molecules at fixed TEs for optimal sensitivity and spectral resolution due to J-evolution and spectral overlap^23,30^. In the literature, the Glu peak was detectable up to a TE of 374 ms^53^, while the Gln peak was detectable up to a TE of 130 ms^23^. Moreover, to the best of our knowledge, the precision of T_2_ measurements, in terms of CRLBs or within-subject CVs, has not been reported.

By utilizing frequency-selective pulses, the proposed technique achieves chemical shift encoding with homonuclear decoupling of the targeted spins in the column (t_1_) dimension and J-refocusing of the targeted spins in the row (t_2_) dimension. The J-evolutions of the targeted spins are refocused at TE = 70 ms by the first editing pulse and refocused again at TE = 157.6 ms by the second editing pulse while undergoing T_2_ decay in the row dimension and maintaining high-amplitude pseudo-singlet spectral structure in the column dimension. Consequently, the dynamic range for measuring the T_2_s of the targeted J-coupled spins is greatly expanded. Although the dataset is acquired in a 2D fashion, no coherence or polarization transfer occurs in the experiment by design. Therefore, a 2D Fourier transform of the acquired dataset is not used. Instead, Fourier transforms are only applied in the column (t_1_) dimension. In the row (t_2_) dimension, all columns are averaged into a small number of bins to increase the SNR of each spectrum and speed up the post-processing procedure for calculating the neurochemical T_2_s. This technique enables the detection of Glu and Gln up to unprecedented long TEs of 458 ms (bin 10) and 220 ms (bin 3), respectively. The very high precision of Glu and Gln T_2_s, as shown in Table 1, makes this technique a viable method for characterizing cellular pathophysiology of glutamatergic neurons and glia in vivo.

For all six healthy participants, the specific absorption rate (SAR) was below 60% of the limit, indicating that the SAR limit does not pose a problem for this sequence at 7 T. As described in the Supplementary Information, the bandwidth of the refocusing pulses is 2.0 kHz. The maximum chemical shift displacement for the neurochemicals of interest, which are the closely resonating Glu, Gln, and GSH glutamyl H4 protons, is only ± 1.7% of the voxel size. Additionally, it is possible to use adiabatic slice-selective pulses in the pulse sequence to further reduce the chemical shift displacement error.

Since the current sequence of the proposed technique is designed to measure the concentrations and T_2_s of Glu, Gln, and GSH, a parsimonious fitting range of 1.8–3.4 ppm is sufficient for reliable quantification of these neurochemicals. The NAA singlet at 2.01 ppm is only 33 Hz away from the 2.12 ppm band of the editing pulses, causing partial dephasing of the NAA singlet. Therefore, NAA was not included in the analysis. Similar to other spectral editing techniques, B_1_ + inhomogeneity can introduce quantification errors in this technique. However, this B_1_ + inhomogeneity issue may be mitigated by using parallel RF transmission (pTx). Neurochemical quantification using this technique is expected to be less affected by line broadening caused by B_0_ inhomogeneity since the targeted J-coupled peaks are pseudo singlets, which are better resolved than multiplets, as shown in Figs. 3, 4 and 5. The shortest TE in our spectra is 71.4 ms for bin 0, which may limit the ability of our method to detect much shorter neurochemical T_2_s. The current implementation of this technique also has a relatively long scan time, which could be reduced in future studies. The high quality of the spectra obtained in this study indicates that the voxel size can also be reduced in future investigations. Adapting this technique to a spectroscopic imaging technique would require an echo planar imaging (EPI) or a similar readout. Furthermore, utilizing this technique at lower magnetic field strengths, such as at 3 T, for detecting Glu, Gln, and GSH T_2_ values is expected to be challenging due to reduced chemical shift dispersion.

Another limitation of the proposed technique is the potential diffusion effect on the measured neurochemical T_2_ values. A rigorous assessment of T_2_ in liquids requires the use of many closely spaced 180° refocusing pulses to minimize the diffusion effect. However, this inevitably generates the T_1_ effect in spatially localized MRS, as the magnetization spends a significant amount of time outside of the transverse plane during the execution of the many long selective refocusing pulses. It should be noted that, for the practical purpose of using Glu and Gln as endogenous cell type-specific markers or contrast agents, excluding diffusion effect may not be necessary or even desirable. This is because neurochemical diffusion abnormality per se may contribute to the differentiation of cellular pathology.

In summary, we have developed a novel MRS technique that overcomes the inherent difficulties in measuring T_2_ of J-coupled neurochemicals in vivo. It achieves chemical shift encoding with frequency-selective decoupling in each column of the acquired 2D dataset. Chemical shift and T_2_ are separately encoded in different dimensions. The columns of the 2D dataset are averaged into a small number of bins with the J-splitting of the targeted spins fully eliminated or suppressed. The background signals in the bin spectra are minimized by T_2_ decay and TE averaging. The dynamic range for measuring the T_2_s of the targeted spins is markedly broadened to 458 ms for Glu and 220 ms for Gln due to the decoupling effect of the editing pulses in the column (t_1_) dimension and the J-refocusing effect of the editing pulses in the row (t_2_) dimension. Furthermore, multi-band editing pulses can be used to decouple multiple molecules and suppress unwanted interfering signals.

As an application of the proposed technique, the concentrations and T_2_ relaxation times of Glu, Gln, and GSH were measured. Bin spectra with well-resolved and sharp Glu, Gln, and GSH peaks were obtained with minimized spectral interference from the NAA aspartyl signals at 2.4–2.6 ppm. Glu and Gln T_2_s were quantified with very high precision. The proposed technique therefore offers a viable approach for noninvasively establishing cell type-specific biomarkers of cellular pathophysiology in neuropsychiatric disorders in vivo.

## Methods

### Proof of concept

**Figure 7:**
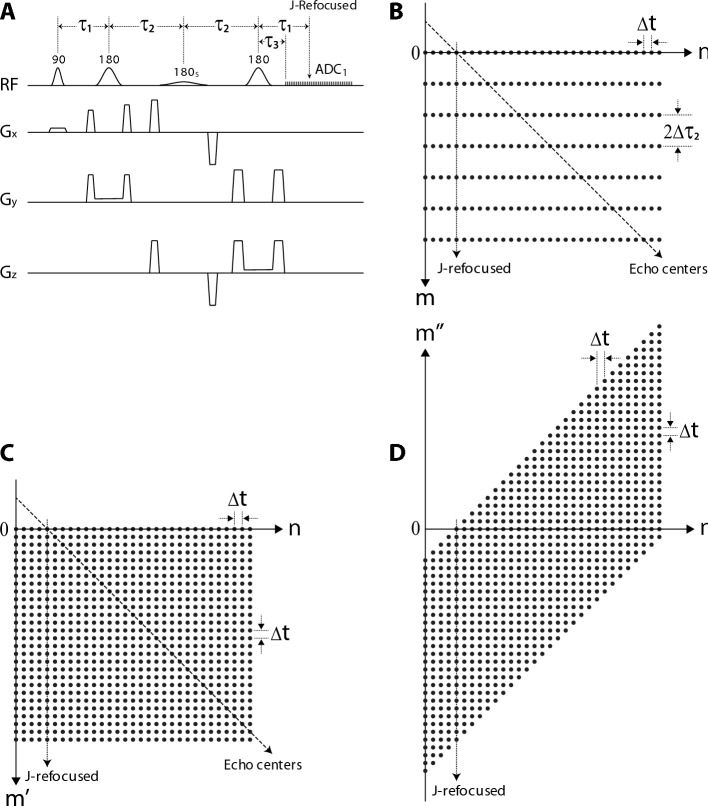
Basic pulse sequence and 2D datasets of the proposed TREND technique. (**A**) Schematic diagram of the basic pulse sequence. The frequency-selective editing pulse is denoted as 180_S_. τ_1_ = 17.5 ms; τ_2_ =  τ_2,0_ + m∆τ_2_, where τ_2,0_ = 17.5 ms, m = 0, 1, … 255, and ∆τ_2_ = 0.4 ms; τ_3_ = 8.3 ms; duration of 180_S_ = 15 ms; dwell time ∆t = 0.2 ms. (**B**) The 2D dataset in the (m, n) space. (**C**) The 2D dataset in the (m’, n) space after sinc-interpolation. (**D**) The 2D dataset in the (m”, n) space after aligning the echo centers.

A schematic diagram of a basic pulse sequence for the proposed technique is shown in Fig. 7A. This sequence is created by adding a 180° frequency-selective editing pulse at the midpoint between the two 180° refocusing pulses of a point-resolved spectroscopy (PRESS) sequence^54^. The editing pulse has a duration of 15 ms and its amplitude profile is generated by truncating a Gaussian function at one standard deviation on each side, leading to a full width half maximum (FWHM) bandwidth of 73 Hz. This editing pulse is applied at 4.10 ppm, targeting the H2 methine proton of lactate, to achieve homonuclear decoupled chemical shift encoding for the H3 protons of lactate at 1.31 ppm. The timing parameters τ_1_, τ_2_, and τ_3_ are labeled in Fig. 7A and their values are: τ_1_ = 17.5 ms, τ_3_ = 8.3 ms, and τ_2_ =  τ_2,0_ + m∆τ_2_, where τ_2,0_ = 17.5 ms, m is the τ_2_ increment number given by m = 0, 1, …, 255, and ∆τ_2_ = 0.4 ms. The data acquisition window ADC_1_ uses a dwell time of 0.2 ms and acquires 1060 data points. The pulse sequence uses 256 τ_2_ increments with fixed τ_1_ and τ_3_.

A 256 × 1060 2D dataset was simulated for 256 τ_2_ increments using the pulse sequence shown in Fig. 7A. A schematic diagram of the timing of the data points is shown in Fig. 7B. The row number m and column number n are labeled using zero-based numbering. The chemical shift encoding time t_CSE_(m, n) for data point (m, n), which is the time delay between the echo center at 4τ_2_ and the nth data point at τ_1_ + 2τ_2_ + τ_3_ + n∆t for the mth τ_2_ increment, is given by:

1 $${\text{t}}_{{{\text{CSE}}}} \left( {{\text{m}},{\text{ n}}} \right) \, = \tau_{{1}} + \tau_{{3}} - {2}\tau_{{{2},0}} + {\text{ n}}\Delta {\text{t}} - {\text{2m}}\Delta \tau_{{2}} .$$

This shows that the column-wise time interval is 2∆τ_2_ (0.8 ms), which is equal to 4∆t.

For efficient data processing, the column-wise resolution is increased by a factor of 4 using sinc interpolation. After sinc interpolation, the dataset size in the (m’, n) space becomes 1024 × 1060 and the time intervals in both dimensions are ∆t, as shown in Fig. 7C. The chemical shift encoding time t_CSE_(m’, n) is the time delay between the echo center at 4τ_2_ and the nth data point at τ_1_ + 2τ_2_ + τ_3_ + n∆t in the m’th row of the sinc-interpolated dataset. It is given by:

2 $${\text{t}}_{{{\text{CSE}}}} \left( {{\text{m'}},{\text{ n}}} \right) = ({\text{n}} - {\text{m'}} - {\text{n}}_{0} )\Delta {\text{t}},$$

where n_0_ = (2τ_2,0_–τ_1_–τ_3_)/∆t = 46. By setting t_CSE_(m’, n) to zero, the equation for echo centers is n–m’–n_0_ = 0, forming the 45° diagonal line shown in Fig. 7C. The row number for the echo center in the nth column is given by:

3 $${\text{m'}}_{{{\text{ec}}}} \left( {\text{n}} \right) = {\text{n}} - {\text{n}}_{0} .$$

The TE value for the echo center in the nth column, which is the time delay between the excitation pulse and the echo center in row m’_ec_(n), is given by:

4 $${\text{TE}}\left( {\text{n}} \right) = {2}\tau_{{1}} + { 2}\tau_{{3}} + {\text{2n}}\Delta {\text{t}}.$$

Each column in the (m’, n) space is then shifted upwards by m’_ec_(n) data points such that the echo centers of all columns lie on the horizonal n-axis. This creates a new dataset in the (m”, n) space, where the new row number m” has both positive and negative values, with the positive direction being upward (see Fig. 7D). Note that the positive m” direction in Fig. 7D corresponds to the negative m’ direction in Fig. 7C. The void data points in Fig. 7D are filled with zeros. In the (m”, n) space, the chemical shift encoding time for data point (m”, n) becomes independent of the column number n and is given by:

5 $${\text{t}}_{{{\text{CSE}}}} \left( {\text{m''}} \right) = {\text{m''}}\Delta {\text{t}}.$$

This shows that the chemical shift information is Fourier-encoded in each column.

Due to the frequency-selective 180° pulse at τ_1_ + τ_2_ targeting spin S of the lactate I_3_S spin system, the J-evolution of spin I is fully refocused at 2τ_1_ + 2τ_2_, which is τ_1_–τ_3_ after the start of data acquisition for all τ_2_ increments. The column number for the data points with fully refocused J-evolution is n_JR_ = (τ_1_–τ_3_)/∆t = 46. Using the product operator formalism^55–57^, the J-modulation factor of spin I at data point (m, n) is:

6 $${\text{f}}_{{{\text{JM}}}} \left( {\text{n}} \right) = {\text{cos}}[\pi {\text{J}}({\text{n}} - {\text{n}}_{{{\text{JR}}}} )\Delta {\text{t}}],$$

indicating a constant J-modulation factor in each column and therefore achieving homonuclear decoupling of the targeted spins in the column dimension.

To improve the SNR and data processing efficiency, the columns of a dataset were averaged into a small number of bins. Each bin spectrum contains a frequency dependent Bloch-Siegert phase shift function^58^ due to the frequency-selective editing pulse. The Bloch-Siegert phase shift function is the same for both row and column dimensions. As a result, the Bloch-Siegert phase shift function for the bin spectra was calculated by density matrix simulation at a single τ_2_ value.

### The second editing pulse

**Figure 8:**
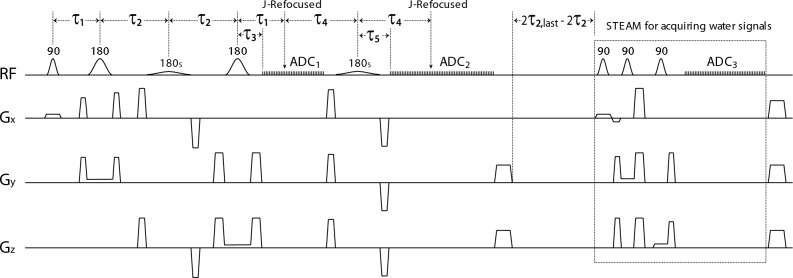
Schematic diagram of the TREND pulse sequence with a second editing pulse (180_S_), a second ADC (ADC_2_), and a STEAM block for acquiring unsuppressed water signals. The time delay between the main sequence block and the STEAM block is 2τ_2,last_–2τ_2_, where τ_2,last_ is the τ_2_ value for the last τ_2_ increment. τ_1_ = 17.5 ms; τ_2_ = τ_2,0_ + m∆τ_2_, where τ_2,0_ = 17.5 ms, m = 0, 1, … 255, and ∆τ_2_ = 0.4 ms; τ_3_ = 8.3 ms; τ_4_ = 21.9 ms; τ_5_ = 10.9 ms; duration of 180_S_ = 15 ms; ADC_1_/ADC_2_/ADC_3_ data points = 100/850/512; and ∆t = 0.2 ms.

As depicted in Fig. 8, a second frequency-selective editing pulse is added to the basic pulse sequence in Fig. 7A. Implementation details of this pulse sequence are provided in the Supplementary Information. The number of data points for ADC_1_ is reduced to 100 and the number of data points for ADC_2_ is set to 850. The values for τ_1_, τ_2_, and τ_3_ values remain the same as in Fig. 7A. The neurochemical signals acquired by ADC_1_ and ADC_2_ are combined and placed into a single 2D dataset in the (m, n) space. The time delay between the start of ADC_1_ and the start of ADC_2_ is τ_1_ + τ_4_ + τ_5_–τ_3_ = 110∆t. Overall, the 2D dataset contains 256 rows and 1060 (100 + 110 + 850) columns, in which columns 100–209 are filled with zeros. J-evolution for the targeted spins in column 265 (210 + (τ_4_–τ_5_)/∆t) is refocused by the 2nd editing pulse.

A stimulated echo acquisition mode (STEAM)^59^ block is appended to the main sequence block to acquire unsuppressed water signals for computing sensitivities of the multi-channel receiver coil^60^ and frequency correction^49^. This STEAM block also pre-saturates the longitudinal magnetizations for the next τ_2_ increment.

### In vivo experiments

Six healthy participants (4 female and 2 male; age = 44 ± 13 years) were recruited and scanned using a Siemens Magnetom 7 T scanner. Written informed consent was obtained from the participants before the study following the procedures approved by the Institutional Review Board (IRB) of the National Institute of Mental Health (NIMH; NCT01266577). All experimental protocols and methods were performed in accordance with the guidelines and regulations of NIH MRI Research Facility. A three-dimensional (3D) T_1_-weighted magnetization prepared rapid gradient echo (MPRAGE) image was acquired with TR = 3 s, TE = 3.9 ms, data matrix = 256 × 256 × 256, and spatial resolution = 1 × 1 × 1 mm^3^. A 2 × 3.5 × 2 cm^3^ MRS voxel was placed in the cingulate cortex with a water linewidth of 13.4 ± 1.6 Hz. The proposed pulse sequence (Fig. 8) was used to acquire data with dual-band editing pulses. These pulses had a 180° band at 2.12 ppm and a 90° band at 4.38 ppm targeting the NAA aspartyl CH proton. Seven variable power RF pulses (sinc-Gauss pulse; duration = 26 ms; FWHM bandwidth = 105 Hz) with optimized relaxation delays (VAPOR) were used for water suppression.

The in vivo dataset in the (m”, n) space was averaged into 11 bins with bin 0 containing all 100 columns of data from ADC_1_ and the remaining 850 columns from ADC_2_ evenly divided by bins 1–10. Details on T_2_ quantification are provided in the Supplementary Information.

## Supplementary Information

Supplementary Information.

## Data Availability

The data acquired during the study and the code developed to analyze the acquired data are available at https://www.nitrc.org/doi/landing_page.php?10.25790/bml0cm.134.
