# Supplementary material for: In vivo magnetic resonance spectroscopy by transverse relaxation encoding with narrowband decoupling
Source: Sci Rep. 2023 Jul 27;13:12211. doi: 10.1038/s41598-023-39375-0 (PMC10374641; doi:10.1038/s41598-023-39375-0)
Supplement: Supplementary file 1 — Supplementary Information. [file 41598_2023_39375_MOESM1_ESM.pdf]

Supplementary Information

In Vivo Magnetic Resonance Spectroscopy by Transverse Relaxation Encoding with Narrowband Decoupling

Li An and Jun Shen

Molecular Imaging Branch, National Institute of Mental Health,

National Institutes of Health, Bethesda, MD

Re-test Spectra

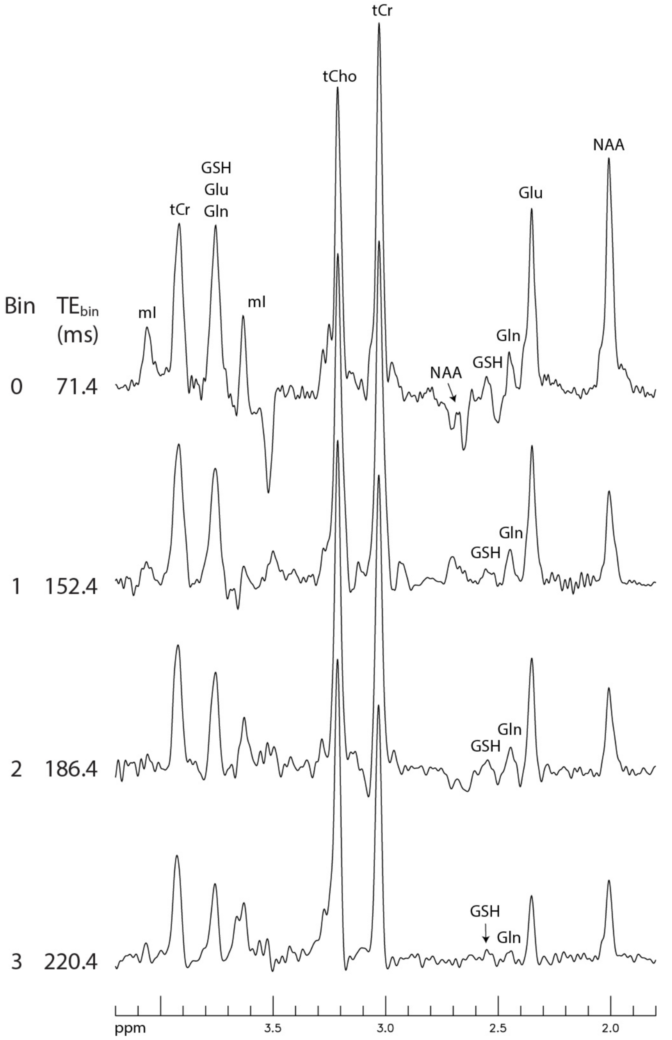

Figure S1. In vivo spectra obtained from the re-test measurement of the spectra shown in Fig. 3.

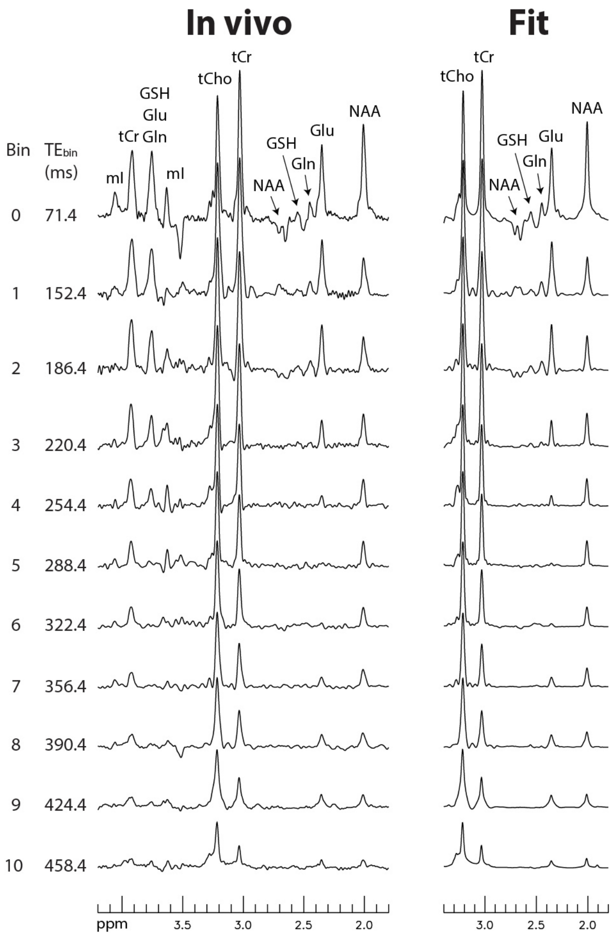

Figure S2. Re-test of Fig. 4 results.

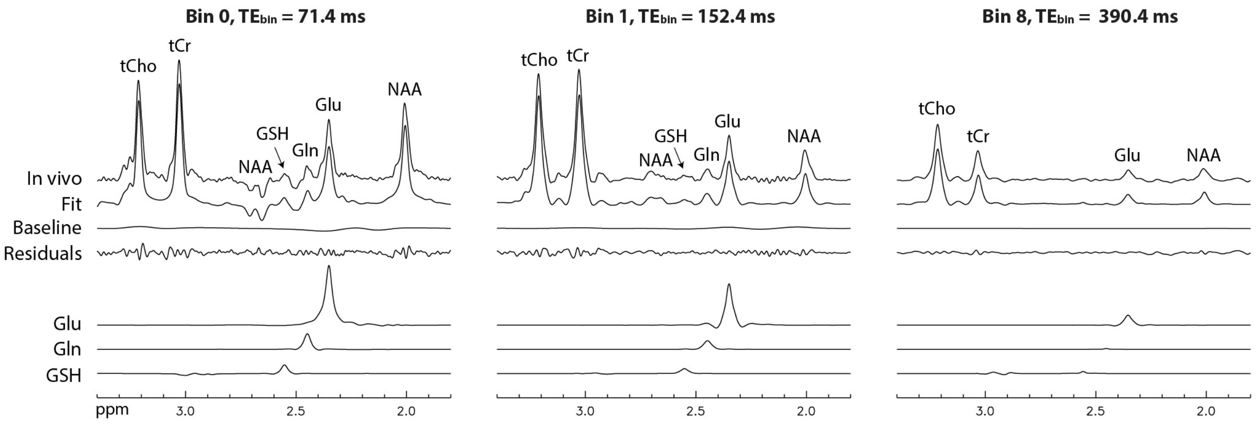

Figure S3. Fitting details for spectra of bins 0, 1, and 8 shown in Fig. S2.

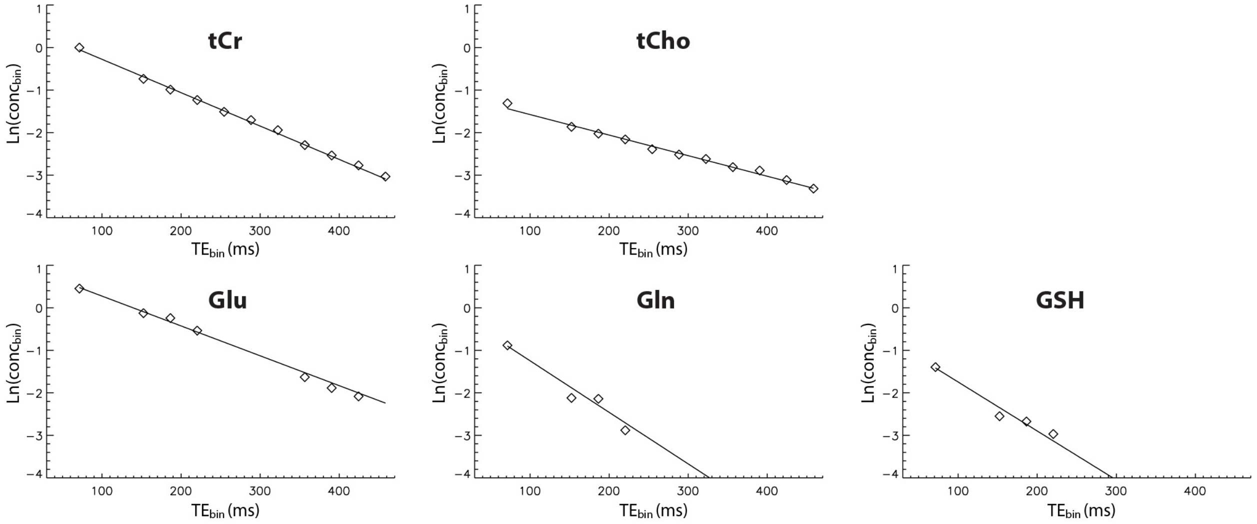

Figure S4. Linear regression analysis of the spectral data shown in Fig. S2.

T_2 Relaxation

The T_2 decay factor for data point (m”, n) is $\exp[-(TE(n) - m''\Delta t)/T_2]$, which can be factorized into $\exp[-(TE(n) - TE_{bin}(h))/T_2] \cdot \exp(m''\Delta t/T_2) \cdot \exp[-TE_{bin}(h)/T_2]$, where $TE_{bin}(h)$ is the average TE of all columns in bin h.

The first factor, $\exp[-(TE(n) - TE_{bin}(h))/T_2]$, corrects for the small intra-bin T_2 decay effect. The second factor, $\exp(m''\Delta t/T_2)$, is independent of the column number and only results in a minor lineshape distortion because $\Delta t \ll T_2$. The third factor, $\exp[-TE_{bin}(h)/T_2]$, represents T_2 decay across different bins and therefore dominates the T_2 decay process. The T_2 and concentration of each molecule were obtained using weighted linear regression analysis[1] with an iterative fitting process. During the first iteration, the first two T_2 decay factors were ignored. Empirically, we found that two or three iterations were sufficient to achieve full convergence in T_2.

A two-sided Voigt function, $R \cdot \exp(-\lambda\,|m''|\Delta t) + (1 - R) \cdot \exp[-a\,(m''\Delta t)^2]$, was multiplied to the basis datasets in the (m”, n) space to account for the line-broadening caused by the reversible T_2’ relaxation effect[2]. Here, R is the proportion of Lorentzian, $\lambda = \pi w$, $a = (\pi w/2)^2 / \ln(2)$, and w is the linewidth.

Numerical Calculation of Basis Functions

Full density matrix simulation with high spatial digitization[3,4], programmed in C++ based on the GAMMA library[5], was used to simulate the proposed pulse sequence for the in vivo experiments. The effects of localization gradients were simulated as frequency shifts of the spin systems[6]. Pre-calculation of propagators[7] and the 1D projection method[3] were used to speed up the computation. Chemical shifts and coupling constants were obtained from Ref.[8] for GABA, from Ref.[9] for GSH, and from Ref.[10] for acetate (Ace), NAA, N-acetylaspartylglutamate (NAAG), Glu, Gln, aspartate (Asp), creatine, phosphocreatine, phosphocholine, glycerophosphocholine, taurine (Tau), myo-inositol (mI), and scyllo-inositol (sI).

Implementation Details of the Proposed Pulse Sequence Shown in Fig. 8

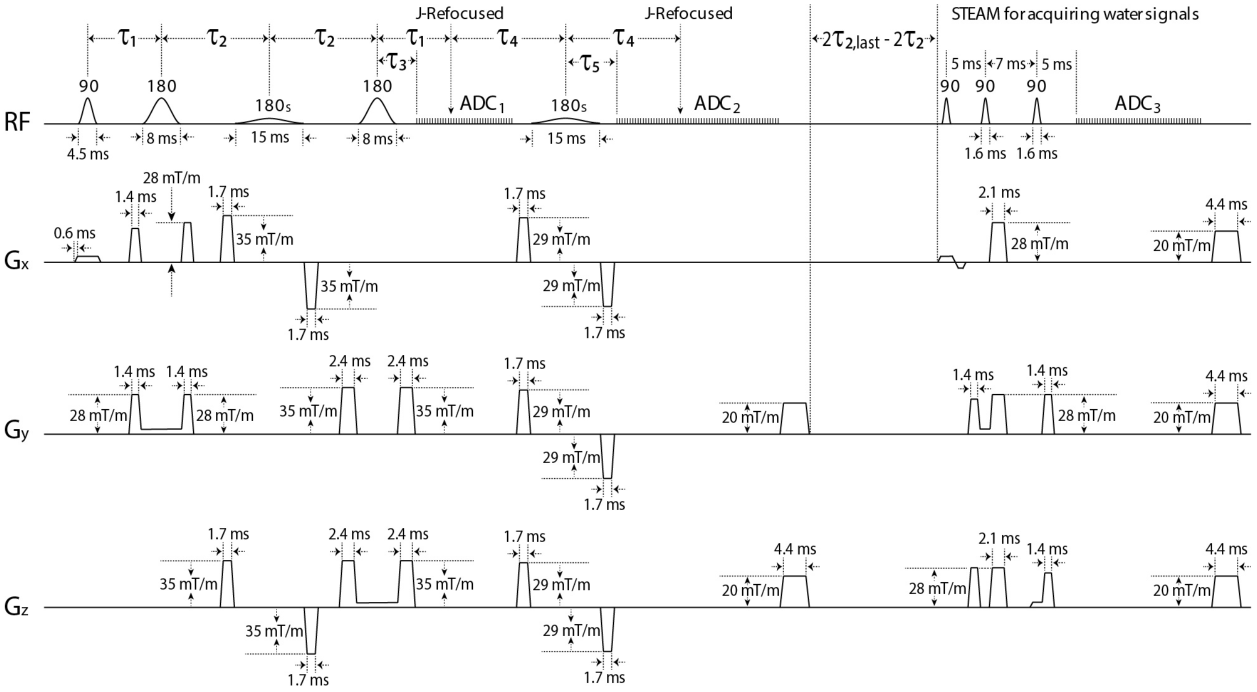

Figure S5. Detailed timing diagram of the TREND pulse sequence shown in Fig. 8.

The slice-selective excitation pulse in the main sequence block was an asymmetric amplitude-modulated pulse[11] with a duration of 4.5 ms, $B_{1,max}$ of 18.6 μT, FWHM bandwidth of 3.1 kHz, and rephase fraction of 0.167. The slice-selective refocusing pulses were amplitude-modulated with a duration of 8.0 ms, $B_{1,max}$ of 18.6 μT, and FWHM bandwidth of 2.0 kHz. The editing pulses (180_S) had a duration of 15 ms and consisted of two bands: a 180° band at 2.12 ppm and a 90° band at 4.38 ppm. The three slice-selective excitation pulses in the STEAM block were a sinc-Gauss pulse with a duration of 1.6 ms, $B_{1,max}$ of 14.9 μT, and FWHM bandwidth of 2.8 kHz. The ramp-up and ramp-down times for all gradients were 0.6 ms. Other used parameters included: τ_1 = 17.5 ms; $\tau_2 = \tau_{2,0} + m\Delta\tau_2$, where $\tau_{2,0}$ = 17.5 ms, m = 0, 1, … 255, and $\Delta\tau_2$ = 0.4 ms; τ_3 = 8.3 ms; τ_4 = 21.9 ms; τ_5 = 10.9 ms; number of data points for $ADC_1/ADC_2/ADC_3$ = 100/850/512; Δt = 0.2 ms; TR = 2.5 s; and total scan time = 10 min and 43 s.

References

1 Chatterjee, S. & Hadi, A. S. *Regression Analysis by Example*. 5th edn, (John Wiley & Sons).

2 Kowalewski, J. & Maler, L. *Nuclear spin relaxation in liquids: Theory, experiments, and applications*. 2nd edn, (CRC Press, 2019).

3 Zhang, Y., An, L. & Shen, J. Fast computation of full density matrix of multispin systems for spatially localized in vivo magnetic resonance spectroscopy. *Medical Physics* **44**, 4169-4178, doi:10.1002/mp.12375 (2017).

4 An, L., Araneta, M. F., Johnson, C. & Shen, J. Effects of carrier frequency mismatch on frequency-selective spectral editing. *Magnetic Resonance Materials in Physics Biology and Medicine* **32**, 237-246, doi:10.1007/s10334-018-0717-5 (2019).

5 Smith, S. A., Levante, T. O., Meier, B. H. & Ernst, R. R. Computer-Simulations in Magnetic-Resonance - an Object-Oriented Programming Approach. *Journal of Magnetic Resonance Series A* **106**, 75-105, doi:DOI 10.1006/jmra.1994.1008 (1994).

6 Kaiser, L. G., Young, K. & Matson, G. B. Numerical simulations of localized high field H-1 MR spectroscopy. *Journal of Magnetic Resonance* **195**, 67-75, doi:10.1016/j.jmr.2008.08.010 (2008).

7 An, L., Li, S. Z., Wood, E. T., Reich, D. S. & Shen, J. N-Acetyl-Aspartyl-Glutamate Detection in the Human Brain at 7 Tesla by Echo Time Optimization and Improved Wiener Filtering. *Magnetic Resonance in Medicine* **72**, 903-912, doi:10.1002/mrm.25007 (2014).

8 Kaiser, L. G., Young, K., Off, D. J. M., Mueller, S. G. & Matson, G. B. A detailed analysis of localized J-difference GABA editing: theoretical and experimental study at 4T. *Nmr in Biomedicine* **21**, 22-32, doi:10.1002/nbm.1150 (2008).

9 Choi, C. H. *et al.* Improvement of resolution for brain coupled metabolites by optimized H-1 MRS at 7 T. *Nmr in Biomedicine* **23**, 1044-1052, doi:Doi 10.1002/Nbm.1529 (2010).

10 Govind, V. H-1-NMR Chemical Shifts and Coupling Constants for Brain Metabolites. *Emagres* **5**, 1347-1362, doi:10.1002/9780470034590.emrstm1530 (2016).

11 Murdoch, J. B., Lent, A. H. & Kritzer, M. R. Computer-Optimized Narrow-Band Pulses for Multislice Imaging. *Journal of Magnetic Resonance* **74**, 226-263, doi:Doi 10.1016/0022-2364(87)90336-2 (1987).
